# Performance Comparison of Repetition Coding MIMO Optical Wireless Communications with Distinct Light Beams

**DOI:** 10.3390/s22031256

**Published:** 2022-02-07

**Authors:** Jupeng Ding, Chih-Lin I, Jintao Wang, Hui Yang, Lili Wang

**Affiliations:** 1 Key Laboratory of Signal Detection and Processing in Xinjiang Uygur Autonomous Region, School of Information Science and Engineering, Xinjiang University, Urumqi 830046, China; 2 China Mobile Research Institute, Beijing 100053, China; icl@chinamobile.com; 3 Department of Electronic Engineering, Beijing National Research Center for Information Science and Technology, Tsinghua University, Beijing 100084, China; wangjintao@tsinghua.edu.cn (J.W.); huiyang@tsinghua.edu.cn (H.Y.); 4 School of Information and Electrical Engineering, Ludong University, Yantai 264025, China; wanglili78@hotmail.com

**Keywords:** repetition coding, MIMO, optical wireless communications, distinct light beams

## Abstract

In current optical wireless communications (OWC) oriented research works, the various multiple input multiple output (MIMO) techniques are introduced and utilized to enhance the coverage performance. Objectively, this Lambertian light beam based MIMO research paradigm neglects the light beam diversity and the potential performance gains. In this work, the distinct non-Lambertian light beams of commercially available light emitting diodes (LEDs) were adopted to configure MIMO OWC links. Specifically, the homogenous and heterogeneous non-Lambertian MIMO configurations were constituted in typical indoor scenarios. Moreover, applying the repetition coding (RC) MIMO algorithm with low complexity, a spatial coverage performance comparison was made between the several above mentioned non-Lambertian configurations and the well-discussed Lambertian MIMO configuration. Numerical results illustrate that the homogeneous NSPW light beam configuration could provide a more than 30 dB average signal to noise ratio (SNR), while the achievable average SNR of the heterogeneous light beam configuration was up to 28.77 dB. On the other side, the counterpart of the Lambertian configuration achieved just about 27.00 dB. Objectively, this work paves the fundamental and essential way for the further design and optimization of MIMO OWC in this novel light beam dimension.

## 1. Introduction

As one vital enabling technology of next generation mobile networks, i.e., beyond 5G or 6G, optical wireless communication (OWC) is earning overwhelming discussion from the academic and research communities [1,2,3,4,5]. More importantly, OWC possesses several unique characteristics, including abundant optical spectrum resources, inherent security, no spectrum regulation, and immunity to severe radio frequency (RF) interference [3,4,5]. Although OWC could reuse ubiquitous illumination infrastructure, the original coverage performance of OWC was severely limited by the spatial beam characteristics of light emitting diodes (LED), which is the key component of OWC transmitters [1,2,3,4].

On the other side, for improving the link reliability and transmission capacity, various multiple input multiple output (MIMO) techniques have been aggressively applied in the OWC domain exploring degree of spatial freedom [6,7,8,9]. Nevertheless, it must be noted that the current MIMO OWC research paradigm usually assumes that the LED sources follow well-discussed Lambertian radiation patterns with quite low complexity.

Thanks to developments in solid state lighting, there are many commercially available LED sources rendering distinct non-Lambertian light beams [10,11,12,13,14]. As a matter of fact, for deriving customized lighting performance, the reflection cups and secondary lens are elaborately attached to the original LED modules by the manufacturers. Some pioneering works have elementarily identified the significant single input single output (SISO) channel characteristics induced by actual non-Lambertian light beams [15,16,17,18]. Specifically, in [15] the non-circular symmetric optical beam was introduced to the cells planning of visible light communication networks. In [16], for focusing more optical signal power to the user terminals, one optical beam-switchable access point was proposed using asymmetrical emission beams. In [17], a modified version of the Monte Carlo-based non-deterministic modeling scheme was systematically proposed for flexibly modeling practical non-Lambertian source radiation patterns in optical wireless channel characterization. In [18], the link coverages of outdoor VLC with actual LED street luminaries were modeled and evaluated. Nevertheless, current non-Lambertian OWC performance analysis is still limited to single output configurations and has not been sufficiently extended to MIMO configurations.

In the following reported work, motivated by the above analysis, the distinct non-Lambertian light beams were further explored in repetition coding (RC) MIMO OWC transmitter configurations. It should be noted that in RC MIMO transmission, the same data stream is sent via each of the transmitters [9]. Moreover, the non-Lambertian light beam based homogeneous and heterogeneous MIMO OWC configurations were proposed, and the relevant indoor coverage performance was modeled and estimated in typical indoor scenarios. In addition, the effect of access point (AP) spacing on coverage performance was estimated under a superior non-Lambertian configuration. It should be noted that, in this work, all concerned non-Lambertian beam characteristics were based on the prestigious and fundamental modeling work for representative and distinct actual LED beams of I. Moreno et al., in [11]. Therefore, the expected sufficient generalization could be satisfied through the whole following reported work.

## 2. Distinct Light Beams Characteristics and Channel Model

### 2.1. General Lambertian Light Beams

**Figure 1:**
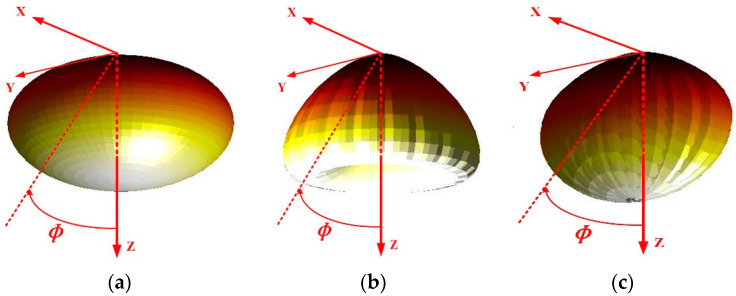
3D spatial radiation pattern from typical view of (**a**) traditional Lambertian light beam, (**b**) LUXEON Rebel light beam, and (**c**) NSPW345CS light beam [11,19].

In current MIMO OWC performance analysis, LED sources usually follow a simple Lambertian light beam pattern. The above pattern assumption means that the radiation intensity is just one cosine function of the viewing angle and could be given as:

(1) $$I_{L}\left(\phi \right)=\frac{m_{L}+1}{2\pi }\cos^{m_{L}}\left(\phi \right),$$

where *m*_L_ is the order of Lambertian emission and ϕ is the irradiance angle relative to the optical axis of the optical source [19]. The Lambertian order *m*_L_ is given by:

(2) $$m_{L}=-\frac{\ln2}{\ln(\cos(\phi_{1/2}))},$$

where $\phi_{1/2}$ is the half power angle of the Lambertian beam [10,19]. Specifically, in Figure 1a, the generalized 3D Lambertian light beam is illustrated for clarity.

### 2.2. Typical Non-Lambertian Light Beams

According to the reported measurement results, the non-Lambertian light beams were numerically fitted. In this work, without a loss of generality, two typical non-Lambertian light beams were considered. Specifically, these light beams were modeled from the LUXEON Rebel LED and the NSPW345CS LED, respectively.

In the LUXEON Rebel light beam case, the spatial radiation intensity could be profiled by the sum of two Gaussian functions [11]:

(3) $$I_{\mathrm{LUX}}(\phi )=\sum_{i=1}^{2}g_{1i}^{\mathrm{LUX}}\exp[-\ln2{\left(\frac{\left|\phi \right|-g_{2i}^{\mathrm{LUX}}}{g_{3i}^{\mathrm{LUX}}}\right)}^{2}],$$

where the coefficient values of Gaussian functions are identified as $g_{11}^{\mathrm{LUX}}$ = 0.76, $g_{21}^{\mathrm{LUX}}$ = 0°, $g_{31}^{\mathrm{LUX}}$ = 29°, $g_{12}^{\mathrm{LUX}}$ = 1.10, $g_{22}^{\mathrm{LUX}}$ = 45°, and $g_{32}^{\mathrm{LUX}}$ = 21°. Correspondingly, the 3D display of LUXEON Rebel LB light beam is shown in Figure 1b.

As for the NSPW345CS case, the spatial radiation intensity should also be profiled by one sum of two Gaussian functions [11]:

(4) $$I_{\mathrm{NSPW}}(\phi ,\alpha )=\sum_{i=1}^{2}g_{1i}^{\mathrm{NSPW}}\exp\left[-\left(\mathrm{In}2\right){\left(\left|\phi \right|-g_{2i}^{\mathrm{NSPW}}\right)}^{2}\left(\frac{\cos^{2}\alpha }{{\left(g_{3i}^{\mathrm{NSPW}}\right)}^{2}}+\frac{\sin^{2}\alpha }{{\left(g_{4i}^{\mathrm{NSPW}}\right)}^{2}}\right)\right],$$

where *α* denotes the azimuth angle, the coefficient values of Gaussian functions are given as $g_{11}^{\mathrm{NSPW}}$ = 0.13, $g_{21}^{\mathrm{NSPW}}$ = 45°, $g_{31}^{\mathrm{NSPW}}$ = $g_{41}^{\mathrm{NSPW}}$ = 18°, $g_{12}^{\mathrm{NSPW}}$ = 1, $g_{22}^{\mathrm{NSPW}}$ = 0, $g_{32}^{\mathrm{NSPW}}$ = 38°, and $g_{42}^{\mathrm{NSPW}}$ = 22°. Similarly, the 3D display of NSPW345CS UB light beam is shown in Figure 1c.

### 2.3. Channel Model

In the well-known Lambertian beam configuration, the OWC direct current (DC) channel gain includes line of sight (LOS) and non-line-of-sight (NLOS) components. In simplified analyses, the NLOS components are usually ignored. Therefore, the respective channel gain could be given as [10,19]:

(5) $$H_{mn}^{\mathrm{Lam}}=\left\{\begin{array}{cc} \frac{A_{R}}{d_{mn}^{2}}\frac{m_{L}+1}{2\pi }\cos^{m_{L}}\left(\phi_{mn}\right)G_{\mathrm{oc}}\cos\left(\theta_{mn}\right), & 0\leq \theta_{mn}\leq \theta_{\mathrm{FOV}}\\ 0, & \theta_{mn}>\theta_{\mathrm{FOV}} \end{array}\right.,$$

where *A_R_* denotes the physical area of the photo detector (PD) of the receiver, *d_mn_* denotes the distance between the *n*th optical access point (AP) and the *m*th PD of the receiver, $G_{\mathrm{oc}}=\frac{n_{\mathrm{RI}}^{2}}{\sin^{2}\left(\theta_{\mathrm{FOV}}\right)}$ denotes the optical concentrator gain at the receiver with an internal refractive index $n_{\mathrm{RI}}$, $\phi_{mn}$ is the irradiance angle from the *n*th optical AP to the *m*th PD, $\theta_{mn}$ is the incidence angle from the *n*th optical AP to the *m*th PD, and *θ*_FOV_ is the field of view (FOV) at the receiver.

Apparently, the OWC DC channel gain is closely related to the optical beam radiation pattern. Respectively, in the LUXEON Rebel beam case, the channel gain between the nth optical AP and the *m*th photo detector of the receiver should be given by:

(6) $$H_{mn}^{\mathrm{LUX}}=\left\{\begin{array}{cc} \frac{A_{R}}{d_{mn}^{2}}\sum_{i=1}^{2}g_{1i}^{\mathrm{LUX}}\exp[-\ln2{\left(\frac{\left|\phi_{mn}\right|-g_{2i}^{\mathrm{LUX}}}{g_{3i}^{\mathrm{LUX}}}\right)}^{2}]G_{\mathrm{oc}}\cos\left(\theta_{mn}\right), & 0\leq \theta_{mn}\leq \theta_{\mathrm{FOV}}\\ 0, & \theta_{mn}>\theta_{\mathrm{FOV}} \end{array}\right.,$$

Similarly, in the NSPW345CS beam case, the DC channel gain should be replaced by:

(7) $$H_{mn}^{\mathrm{NSPW}}=\left\{\begin{array}{ll} \frac{A_{R}}{d_{mn}^{2}}\sum_{i=1}^{2}g_{1i}^{\mathrm{NSPW}}\exp\left[-\left(\ln2\right){\left(\left|\phi_{mn}\right|-g_{2i}^{\mathrm{NSPW}}\right)}^{2}\left(\frac{\cos^{2}\alpha_{mn}}{{\left(g_{3i}^{\mathrm{NSPW}}\right)}^{2}}+\frac{\sin^{2}\alpha_{mn}}{{\left(g_{4i}^{\mathrm{NSPW}}\right)}^{2}}\right)\right]G_{\mathrm{oc}}\cos\left(\theta_{mn}\right), & 0\leq \theta_{mn}\leq \theta_{\mathrm{FOV}}\\ 0, & \theta_{mn}\leq \theta_{\mathrm{FOV}} \end{array}\right.,$$

where *α*_mn_ denotes the azimuth angle of the *m*th photo detector to the nth optical AP.

## 3. Repetition Coding MIMO OWC Transmission System

**Figure 2:**
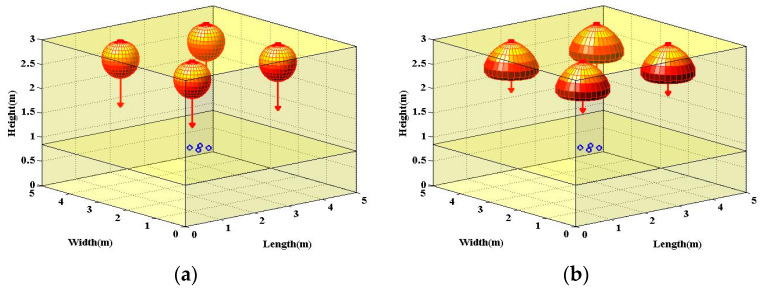

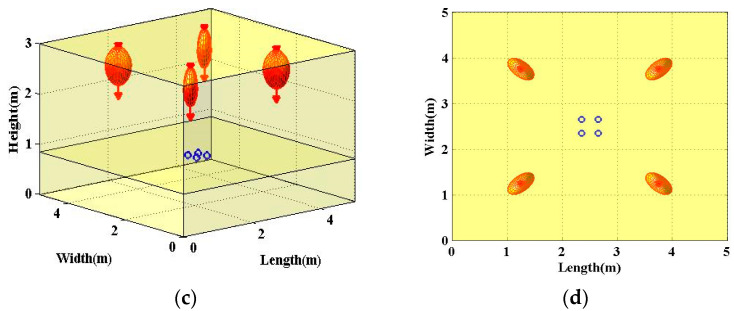
×4 MIMO optical wireless communications scenario adopting: (**a**) distributed traditional Lambertian light beam configuration, (**b**) distributed LUXEON Rebel light beam configuration, and (**c**) distributed NSPW345CS light beam configuration from typical view. Panel (**d**) is the top view of the last configuration for clarity of the rotational asymmetry of NSPW345CS light beams. Each blue circle denotes one PD of the receiver.

For one typical MIMO OWC system with four distributed APs and one receiver with four PDs, the output signal vector of the receiver is described by [8,9]:

(8) y=Hx+n,

where **x** = [x_1_, x_2_, x_3_, x_4_] are emitted symbols from the four APs, **y** = [y_1_, y_2_, y_3_, y_4_] are the received symbols at the receiver, **n** = [n_1_, n_2_, n_3_, n_4_] are the additive white Gaussian noise (AWGN) values at the PDs. If the well-known homogeneous Lambertian light beam configuration is adopted in this MIMO OWC system, the channel gain matrix **H** is given by:

(9) $$H=\left(\begin{array}{cccc} H_{11}^{\mathrm{Lam}} & H_{12}^{\mathrm{Lam}} & H_{13}^{\mathrm{Lam}} & H_{14}^{\mathrm{Lam}}\\ H_{21}^{\mathrm{Lam}} & H_{22}^{\mathrm{Lam}} & H_{23}^{\mathrm{Lam}} & H_{24}^{\mathrm{Lam}}\\ H_{31}^{\mathrm{Lam}} & H_{32}^{\mathrm{Lam}} & H_{33}^{\mathrm{Lam}} & H_{34}^{\mathrm{Lam}}\\ H_{41}^{\mathrm{Lam}} & H_{42}^{\mathrm{Lam}} & H_{43}^{\mathrm{Lam}} & H_{44}^{\mathrm{Lam}} \end{array}\right),$$

where the element $H_{ij}^{\mathrm{Lam}}$ of **H** could be calculated specifically via (5). Under such a homogeneous Lambertian light beam configuration, the typical indoor scenario is shown in Figure 2a.

If the all Lambertian light beams are replaced by the LUXEON Rebel light beams, the respective non-Lambertian homogeneous MIMO OWC configuration is constituted. Accordingly, the channel gain matrix **H** is renewed by:

(10) $$H=\left(\begin{array}{cccc} H_{11}^{\mathrm{LUX}} & H_{12}^{\mathrm{LUX}} & H_{13}^{\mathrm{LUX}} & H_{14}^{\mathrm{LUX}}\\ H_{21}^{\mathrm{LUX}} & H_{22}^{\mathrm{LUX}} & H_{23}^{\mathrm{LUX}} & H_{24}^{\mathrm{LUX}}\\ H_{31}^{\mathrm{LUX}} & H_{32}^{\mathrm{LUX}} & H_{33}^{\mathrm{LUX}} & H_{34}^{\mathrm{LUX}}\\ H_{41}^{\mathrm{LUX}} & H_{42}^{\mathrm{LUX}} & H_{43}^{\mathrm{LUX}} & H_{44}^{\mathrm{LUX}} \end{array}\right),$$

where the element $H_{ij}^{\mathrm{LUX}}$ of **H** could be calculated specifically by (6). Under such a homogeneous LUXEON Rebel light beams configuration, the typical indoor scenario is shown in Figure 2b.

Similarly, as for the NSPW345CS light beam, the respective non-Lambertian homogeneous MIMO OWC configuration could be constituted as well. In this case, the channel gain matrix **H** is given by:

(11) $$H=\left(\begin{array}{cccc} H_{11}^{\mathrm{NSPW}} & H_{12}^{\mathrm{NSPW}} & H_{13}^{\mathrm{NSPW}} & H_{14}^{\mathrm{NSPW}}\\ H_{21}^{\mathrm{NSPW}} & H_{22}^{\mathrm{NSPW}} & H_{23}^{\mathrm{NSPW}} & H_{24}^{\mathrm{NSPW}}\\ H_{31}^{\mathrm{NSPW}} & H_{32}^{\mathrm{NSPW}} & H_{33}^{\mathrm{NSPW}} & H_{34}^{\mathrm{NSPW}}\\ H_{41}^{\mathrm{NSPW}} & H_{42}^{\mathrm{NSPW}} & H_{43}^{\mathrm{NSPW}} & H_{44}^{\mathrm{NSPW}} \end{array}\right),$$

where the element $H_{ij}^{\mathrm{NSPW}}$ of **H** could be specifically calculated by (7). For clarity, the typical indoor scenario of this homogeneous non-Lambertian configuration is shown in Figure 2c,d from the side and top view, respectively. As shown in Figure 2d, the original azimuth angle of four APs is 45°, 135°, 225°, and 315°, respectively.

Following the work of Harald Haas [9], for convenience, the signal to noise ratio (SNR) of MIMO OWC applying the same repetition coding (RC) MIMO algorithm with low complexity could be calculated as:

(12) $${\mathrm{SNR}}_{\mathrm{MRC}}=\frac{{\left(rI_{\mathrm{TX}}\right)}^{2}}{N_{0}N_{t}^{2}}\sum_{i=1}^{N_{r}}{\left(\sum_{j=1}^{N_{t}}H_{ij}\right)}^{2},$$

where *r* denotes the responsivity of the PDs, *I*_TX_ denotes the emitted power of all transmitters, *N*_0_ denotes the noise power, *N_t_* = 4 denotes the number of the APs, and *N_r_* = 4 denotes the number of the PDs of the receiver. In a well-lit environment, the shot noise is one dominant contributor to the signal disturbance at the receiver. Then, the noise power could be calculated as [19]:

(13) $$N_{0}=2qI_{\mathrm{bg}}B+\frac{4K_{b}TB}{R_{f}},$$

where *q* denotes the electron charge in coulombs, *I*_bg_ denotes the current due to the background light, *B* denotes the system modulation bandwidth, *K*_b_ denotes the Boltzmann constant, *T* denotes the absolute temperature and *R*_f_ denotes the feedback resistance of the transimpedance amplifier (TIA) [19].

**Figure 3:**
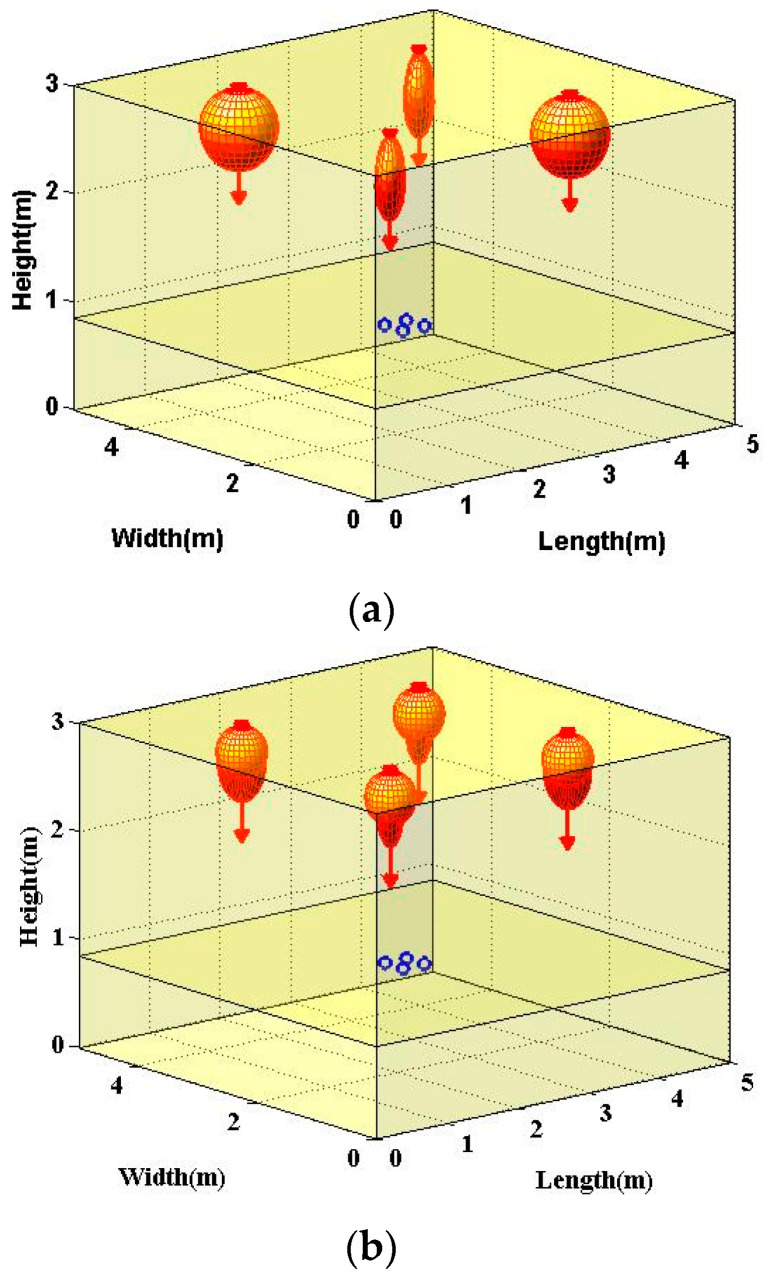
Typical indoor heterogeneous 4 × 4 MIMO optical wireless communications scenario adopting: (**a**) inter-AP heterogeneous light beam configuration, (**b**) intra-AP heterogeneous light beam configuration. Each blue circle denotes one PD of the receiver.

As for the potential heterogeneous light beams configurations, two specific configurations were investigated. Firstly, for the inter-AP heterogeneous light beam configuration, the NSPW345CS non-Lambertian light beams were utilized to replace part of the Lambertian homogeneous configuration. Specifically, in the investigated MIMO OWC system with distributed APs, the two APs with non-Lambertian light beams were placed along the diagonal of the ceiling, as shown in Figure 3a. Typically, the emitted power of each LED chip, i.e., LED element, was quite limited. To satisfy the general lighting function, the LED source, also known as the LED array, is usually composed of numerous LED elements. For the intra-AP heterogeneous configuration, the LED elements with distinct beam characteristics were adopted to constitute the LED array. For convenience of analysis, two types of LED elements were included in the investigated intra-AP heterogeneous configuration. For consistency with the abovementioned homogeneous NSPW345CS configuration, the original azimuth angle of the two NSPW345CS APs was 45° and 225°, respectively. Moreover, the MIMO OWC channel gain matrix of this inter-AP heterogeneous configuration could be given as:

(14) $$H=\left(\begin{array}{cccc} H_{11}^{\mathrm{Lam}} & H_{12}^{\mathrm{NSPW}} & H_{13}^{\mathrm{NSPW}} & H_{14}^{\mathrm{Lam}}\\ H_{21}^{\mathrm{Lam}} & H_{22}^{\mathrm{NSPW}} & H_{23}^{\mathrm{NSPW}} & H_{24}^{\mathrm{Lam}}\\ H_{31}^{\mathrm{Lam}} & H_{32}^{\mathrm{NSPW}} & H_{33}^{\mathrm{NSPW}} & H_{34}^{\mathrm{Lam}}\\ H_{41}^{\mathrm{Lam}} & H_{42}^{\mathrm{NSPW}} & H_{43}^{\mathrm{NSPW}} & H_{44}^{\mathrm{Lam}} \end{array}\right),$$

where the element $H_{ij}^{\mathrm{Lam}}$ and $H_{ij}^{\mathrm{NSPW}}$ of **H** could be specifically calculated by Equations (5) and (7), respectively. Secondly, for the intra-AP heterogeneous light beams configuration, the NSPW345CS non-Lambertian light beam was added to each AP of the above Lambertian homogeneous configuration, as shown in Figure 3b. For a fair comparison, the emitted optical power for each AP was equally divided between the included NSPW345CS and Lambertian light beams. Essentially, in this intra-AP heterogeneous configuration, the MIMO channel gain matrix **H** had to be renewed as:

(15) $$H=0.5\left(\begin{array}{cccc} \left(H_{11}^{\mathrm{Lam}},+,H_{11}^{\mathrm{NSPW}}\right) & \left(H_{12}^{\mathrm{Lam}},+,H_{12}^{\mathrm{NSPW}}\right) & \left(H_{13}^{\mathrm{Lam}},+,H_{13}^{\mathrm{NSPW}}\right) & \left(H_{14}^{\mathrm{Lam}},+,H_{14}^{\mathrm{NSPW}}\right)\\ \left(H_{21}^{\mathrm{Lam}},+,H_{21}^{\mathrm{NSPW}}\right) & \left(H_{22}^{\mathrm{Lam}},+,H_{22}^{\mathrm{NSPW}}\right) & \left(H_{23}^{\mathrm{Lam}},+,H_{23}^{\mathrm{NSPW}}\right) & \left(H_{24}^{\mathrm{Lam}},+,H_{24}^{\mathrm{NSPW}}\right)\\ \left(H_{31}^{\mathrm{Lam}},+,H_{31}^{\mathrm{NSPW}}\right) & \left(H_{32}^{\mathrm{Lam}},+,H_{32}^{\mathrm{NSPW}}\right) & \left(H_{33}^{\mathrm{Lam}},+,H_{33}^{\mathrm{NSPW}}\right) & \left(H_{34}^{\mathrm{Lam}},+,H_{34}^{\mathrm{NSPW}}\right)\\ \left(H_{41}^{\mathrm{Lam}},+,H_{41}^{\mathrm{NSPW}}\right) & \left(H_{42}^{\mathrm{Lam}},+,H_{42}^{\mathrm{NSPW}}\right) & \left(H_{43}^{\mathrm{Lam}},+,H_{43}^{\mathrm{NSPW}}\right) & \left(H_{44}^{\mathrm{Lam}},+,H_{44}^{\mathrm{NSPW}}\right) \end{array}\right)$$

where the element 0.5 × ($H_{ij}^{\mathrm{Lam}}$+$H_{ij}^{\mathrm{NSPW}}$) of **H** could be calculated specifically with reference to $H_{ij}^{\mathrm{Lam}}$ in Equation (5) and to $H_{ij}^{\mathrm{NSPW}}$ in Equation (7).

## 4. Numerical Evaluation

For the following section, the SNR spatial distribution of the discussed homogeneous and heterogeneous MIMO OWC system was evaluated and compared. In addition, for a fair comparison, all concerned light beam intensities were normalized within the following reported work. Furthermore, Table 1 summarizes the main parameters for this work [10,11,19].

**Figure 4:**
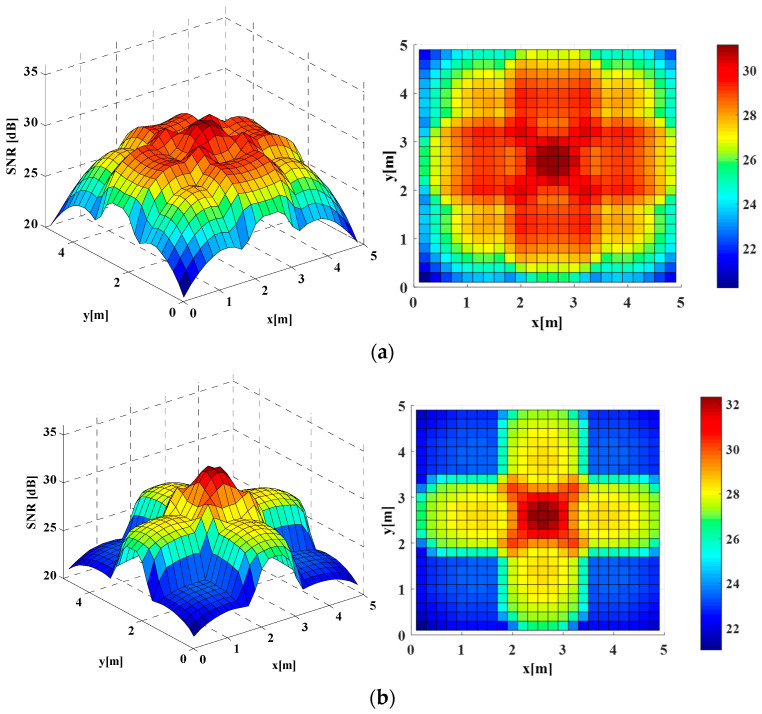

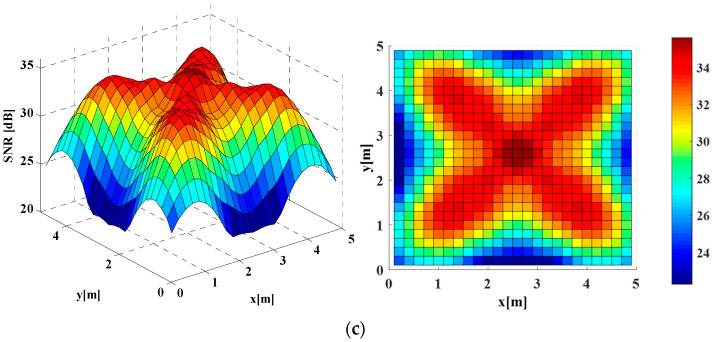
3D and 2D SNR spatial distribution in the case of (**a**) homogeneous distributed Lambertian light beam configuration; (**b**) homogeneous distributed LUXEON Rebel light beam configuration and (**c**) homogeneous distributed NSPW345CS light beam configuration.

It should be noted that all the following results were obtained from simulations and the average operation of results was avoided using the emitted average power and noise statistical variation. As shown in Figure 4a, for the conventional homogeneous Lambertian light beam configuration, the MIMO achievable SNR spatial dynamic range was about 20.23–31.15 dB while the average SNR of this conventional configuration was about 27.00 dB.

On the other side, in the homogeneous distributed LUXEON Rebel light beam MIMO configuration, the achievable SNR spatial dynamic range was reduced to about 21.03–32.35 dB, while the average SNR of this non-Lambertian configuration was reduced to about 25.62 dB, as shown in Figure 4b. Compared to the above Lambertian configuration, an up to 1.38 dB average SNR loss was induced by this homogeneous LUXEON Rebel MIMO configuration.

By contrast, a significant SNR enhancement could be provided by the homogeneous NSPW345CS light beam MIMO configuration. In particular, the respective achievable SNR spatial dynamic range was elevated to about 22.27–35.61 dB, while the average SNR of this non-Lambertian configuration was increased to 30.10 dB, as shown in Figure 4c. Therefore, an up to about 3.10 dB average SNR gain was obtained by this homogeneous NSPW345CS MIMO configuration, compared to the previous Lambertian configuration. For all compared three homogeneous configuration, the SNR spatial distributions presented intuitionally axial symmetry. In addition, the respective maximum MIMO SNR gain was up to 4.46 dB greater compared to the homogeneous Lambertian configuration.

**Figure 5:**
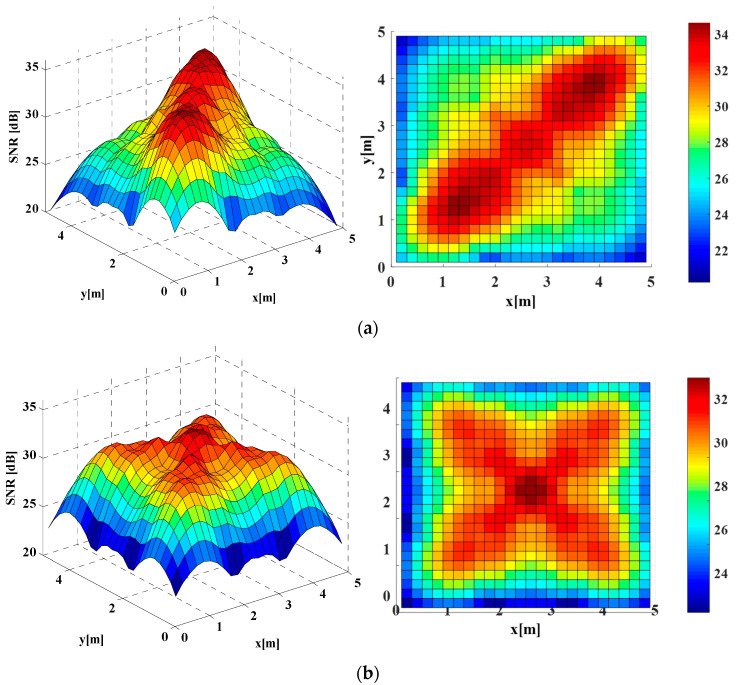
3D and 2D SNR spatial distribution in the case of (**a**) inter-AP heterogeneous light beams configuration; (**b**) intra-AP heterogeneous light beams configuration.

As for the proposed inter-AP heterogeneous light beam configuration, the SNR spatial distribution presented obvious asymmetry, as shown in Figure 5a. In this situation, SNR peaks of about 34.64 dB appeared at the receivers under the two NSPW345CS APs on the receiver plane. The average SNR of this inter-AP heterogeneous configuration was about 28.58 dB; the gain was about 1.58 dB compared to the original Lambertian configuration.

Unlike the above inter-AP heterogeneous configuration, the SNR spatial distribution of the intra-AP heterogeneous configuration did not exhibit asymmetry anymore, as shown in Figure 5b. In this case, the respective achievable SNR spatial dynamic range was changed to about 22.89–33.65 dB, while the average SNR of this intra-AP heterogeneous configuration was increased to 28.77 dB. Correspondingly, the achieved average SNR gain was about 1.77 dB compared to the benchmark Lambertian configuration. Moreover, the respective maximum receiver SNR was 33.65 dB, while an up to 2.50 dB maximum MIMO coverage SNR gain could be derived from this light beam heterogeneity.

**Figure 6:**
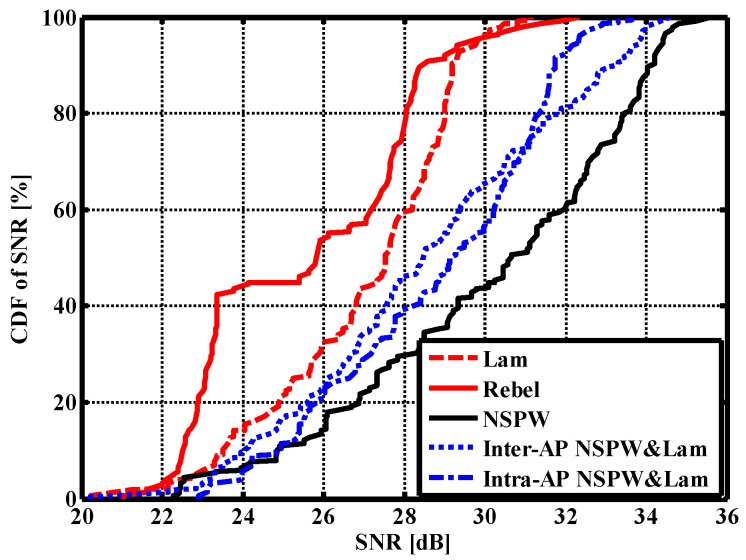
SNR cumulative distribution function of discussed homogeneous and heterogeneous MIMO optical wireless communications scenarios.

To compare the statistical characteristics of SNR distributions in the three homogeneous configurations and two heterogeneous configurations considered, the SNR cumulative distribution function (CDF) curves are described in Figure 6. For up to 80% of the receiver positions in the Lambertian configuration, the SNR was more than 24.87 dB, while the counterparts of the homogeneous LUXEON Rebel configuration and the homogeneous NSPW345CS configurations changed to 22.89 dB and 26.88 dB, respectively. Therefore, the proposed homogeneous NSPW345CS configuration is capable of dramatically improving the weak coverage domain in the indoor application scenario.

As for the proposed inter-AP and intra-AP heterogeneous light beam configurations, in up to 80% of receiver positions the SNR was more than 25.58 dB and 25.67 dB, respectively. Therefore, there is an obvious performance tradeoff between the original Lambertian and NSPW345CS light beam homogeneous configurations.

**Figure 7:**
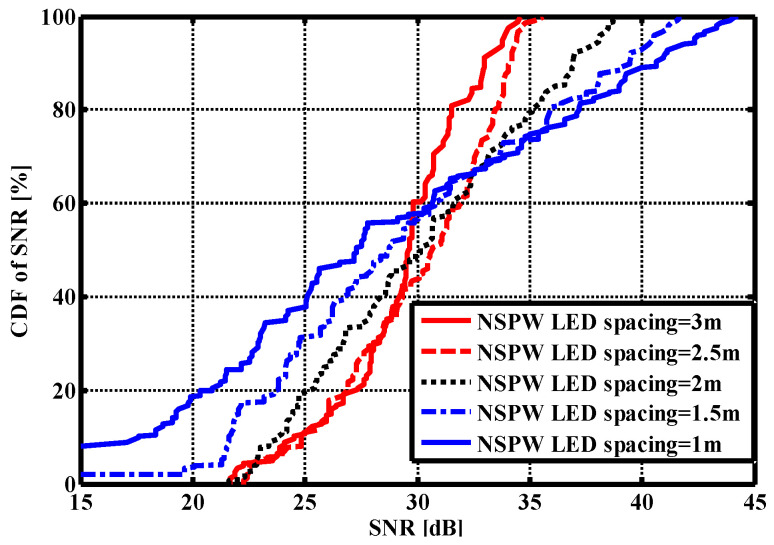
SNR cumulative distribution function with different inter-AP spacing settings.

For identifying the AP spacing effect on the SNR CDF curve, the spacing was reduced from 3 m to 1 m with a 0.5 m step in the NSPW345CS homogeneous configuration. It should be noted that the size of the environment was always the same during the investigations of different LED APs spacing settings. Under different APs spacing settings, the LED APs were always on the vertices of a square centered on the geometrical center of the ceiling. As shown in Figure 7, the fluctuation extent increased to more than 40 dB at the 1 m AP spacing compared to the original 12.88 dB at the 3 m AP spacing. At the same time, the maximum SNR gradually increased to 44.30 dB from 34.56 dB.

## 5. Conclusions

The coverage performance of MIMO VLC systems based on Lambertian and non-Lambertian light beams was evaluated in typical indoor scenarios. This investigation shows that the different light beam configurations could significantly influence the MIMO SNR spatial distribution. As for the intra-AP heterogeneous light beam configuration, the mean SNR was enhanced to about 28.77 dB from about 27.00 dB in the conventional homogeneous Lambertian light beam configuration. Moreover, an up to 2.50 dB maximum MIMO coverage SNR gain could be derived from this light beam heterogeneity, while the counterpart of the homogeneous NSPW light beam configuration further increased to 4.46 dB.

## Figures and Tables

**Table 1:** The main parameters of the configuration.

Parameters	Values
Room size (W × L × H)	5 × 5 × 3 m^3^
Emitted power of all APs	1 W
LED APs spacing	2.5 m
LED Lambertian index	1
Receiver field of view	45°
PD spacing	10 cm
Height of receiving plane	0.85 m
Physical area of PD	1 cm^2^
Responsivity of PD	0. 28 A/W
Concentrator refractive index	1.54
Optical filter gain	1
Modulation bandwidth	20 MHz
Background light current	5100 μA
Absolute temperature	298 K
Feedback resistance of TIA	6 kΩ

## Data Availability

Not applicable.

## References

[1] Song J., Wang X., Wang J., Zhang H., Pan C., Zhang Y., Cosmas J. (2020). The Converged Internet of Lights Network for Telecommunication, Positioning, Illumination, and Medical Therapy. IEEE Commun. Stand. Mag..

[2] Shi L., Shi D., Zhang X., Meunier B., Zhang H., Wang Z., Vladimirescu A., Li W., Zhang Y., Cosmas J. (2020). 5G Internet of Radio Light Positioning System for Indoor Broadcasting Service. IEEE Trans. Broadcast..

[3] Strinati E.C., Barbarossa S., Gonzalez-Jimenez J.L., Ktenas D., Cassiau N., Maret L., Dehos C. (2019). 6G: The Next Frontier: From Holographic Messaging to Artificial Intelligence Using Subterahertz and Visible Light Communication. IEEE Veh. Technol. Mag..

[4] Meunier B., Cosmas J., Jawad N., Ali K. (2020). Realising a new generation of 5G VR systems through internet of radio light. Proceedings of the 2020 IEEE International Symposium on Broadband Multimedia Systems and Broadcasting (BMSB), Paris, France, 27–29 October 2020.

[5] Ma X., Gao J., Yang F., Ding W., Yang H., Song J. (2017). Integrated power line and visible light communication system compatible with multi-service transmission. IET Commun..

[6] Mana S.M., Kouhini S.M., Hellwig P., Hilt J., Berenguer P.W., Jungnickel V. (2020). Distributed MIMO Experiments for LiFi in a Conference Room. Proceedings of the 2020 12th International Symposium on Communication Systems, Networks and Digital Signal Processing (CSNDSP), Online, 20–22 July 2020.

[7] Kouhini S.M., Mana S.M., Freund R., Jungnickel V., Correa C.R.B., Tangdiongga E., Cunha T., Deng X., Linnartz J.-P.M.G. (2020). Distributed MIMO Experiment Using LiFi Over Plastic Optical Fiber. Proceedings of the 2020 IEEE Globecom Workshops (GC Wkshps), Taipei City, Taiwan, 7–11 December 2020.

[8] Tsonev D., Sinanovic S., Haas H. (2013). Practical MIMO Capacity for Indoor Optical Wireless Communication with White LEDs. Proceedings of the 2013 IEEE 77th Vehicular Technology Conference (VTC Spring), Dresden, Germany, 2–5 June 2013.

[9] Fath T., Haas H. (2012). Performance Comparison of MIMO Techniques for Optical Wireless Communications in Indoor Environments. IEEE Trans. Commun..

[10] Ding J., Chih-Lin I., Xu Z. (2016). Indoor optical wireless channel characteristics with distinct source radiation patterns. IEEE Photonics J..

[11] Moreno I., Sun C.-C. (2008). Modeling the radiation pattern of LEDs. Opt. Express.

[12] Moreno I. (2006). Spatial distribution of LED radiation. Proceedings of the International Optical Design Conference 2006, Vancouver, BC, Canada, 17 July 2006.

[13] Yang C., Wang Y.-H., Ding Z., Wen J., Bian L.-F. (2019). Radiation Pattern Measurement in the Planar Coordinate. IEEE Photonics J..

[14] Sun C.-C., Lee T.-X., Ma S.-H., Lee Y.-L., Huang S.-M. (2006). Precise optical modeling for LED lighting verified by cross correlation in the midfield region. Opt. Lett..

[15] Ding J., Chih-Lin I., Zhang H., Chen X., Yu B., Lai H. (2019). Cells Planning of VLC Networks using Non-Circular Symmetric Optical Beam. Proceedings of the ICC 2019—2019 IEEE International Conference on Communications (ICC), Shanghai, China, 20–24 May 2019.

[16] Ding J., Chih-Lin I., Chen X., Lai H. (2019). Asymmetrical Emission Beams based Visible Light Communication Access Points Design. Proceedings of the 2019 28th Wireless and Optical Communications Conference (WOCC), Beijing, China, 9–10 May 2019.

[17] Ding J., Chih-Lin I., Xie R., Lai H., Zhang C. (2018). Actual radiation patterns-oriented non-deterministic optical wireless channel characterization. Proceedings of the Chinese Conference on Biometric Recognition, Urumchi, China, 11–12 August 2018.

[18] Ding J., Chih-Lin I., Zhang C., Yu B., Lai H. (2018). Evaluation of Outdoor Visible Light Communications Links Using Actual LED Street Luminaries. Proceedings of the Chinese Conference on Biometric Recognition, Urumchi, China, 11–12 August 2018.

[19] Komine T., Nakagawa M. (2004). Fundamental analysis for visible-light communication system using LED lights. IEEE Trans. Consum. Electron..

